# Feasibility and tolerability of combining 6 Hz transcranial alternating current stimulation (tACS) at 3.2 mA and digital cognitive training in older adults with mild cognitive impairment

**DOI:** 10.1007/s10072-026-09524-0

**Published:** 2026-11-05

**Authors:** Brunno Freitas da Costa, Karin Reuwsaat, Nwabunwanne Emele, Bruno Costa Poltronieri, Cíntia Monteiro Carvalho, Yasmin Guedes de Oliveira, João Vitor Soares, Ana Carolina Madeira, Amanda Gomes de Azevedo, Rogério Panizzutti

**Affiliations:** 1 Instituto de Psiquiatria, Universidade Federal do Rio de Janeiro, Avenida Venceslau Brás 71, fundos, Praia Vermelha, Rio de Janeiro, RJ 22290140 Brazil; 2 Instituto de Ciências Biomédicas, Universidade Federal do Rio de Janeiro, Rio de Janeiro, Brazil; 3 Instituto Federal de Educação, Ciência e Tecnologia do Rio de Janeiro, Rio de Janeiro, Brazil

**Keywords:** Mild cognitive impairment, Electric stimulation, Cognitive dysfunction, Cognitive training

## Abstract

**Purpose:**

Transcranial alternating current stimulation (tACS) may enhance cognition and potentiate digital cognitive training (DCT) in mild cognitive impairment (MCI). Optimal intensity remains uncertain because higher currents may increase cortical electric fields but raise tolerability concerns. We evaluated feasibility and tolerability of 3.2 mA tACS plus DCT, and exploratorily assessed active versus sham effects on inhibitory control and cognitive transfer.

**Methods:**

Ten older adults with MCI received five daily 6-Hz tACS sessions over the left dorsolateral prefrontal cortex during DCT; ten sham-arm participants from a randomized trial completed identical DCT. Feasibility and tolerability were assessed by adherence, attrition, and side-effect monitoring. Exploratory efficacy used repeated-measures ANOVA with Bonferroni-corrected paired comparisons. Outcomes included antisaccade total errors, verbal and visual memory, and visuoconstructive abilities.

**Results:**

All participants completed treatment (100% adherence). Active-session side effects were mild in 67% and moderate in 23%; no severe events occurred. Side-effect burden was unrelated to DCT performance (*p* = 0.22). Antisaccade total errors showed a group × time interaction (*p* = 0.028), favoring active stimulation. Reaction time showed no stimulation-specific effects. Verbal and visual memory showed time effects (*p* = 0.016 and *p* = 0.002), whereas visuoconstructive abilities showed a trend-level group × time interaction favoring active stimulation (*p* = 0.054).

**Conclusions:**

Higher-intensity tACS plus DCT was feasible and well tolerated in older adults with MCI. Exploratory findings suggest that active stimulation may improve inhibitory control and facilitate transfer to selected cognitive domains. Confirmation in the ongoing randomized controlled trial is warranted.

## Introduction

Cognitive decline in older adults can profoundly impact individual autonomy and places an increasing burden on healthcare systems worldwide [1]. While some degree of cognitive decline may be expected during normal aging, Mild Cognitive Impairment (MCI) marks a more pronounced decline, affecting cognitive processes such as memory and visuospatial abilities, as well as neurophysiological processes like ocular motor control [2]. Moreover, MCI is reported to affect approximately 15% of individuals over 65 years old and represents a condition of increased risk for developing dementia [3], making it an important target population for interventions aimed at preserving cognition before more substantial functional decline develops.

Digital cognitive training (DCT) provides adaptive and repeated practice of specific cognitive processes and can be delivered through accessible and individually adjusted platforms. Meta-analytic and clinical-trial evidence indicates that DCT can improve global cognition and selected cognitive domains in individuals with MCI [4–6]. Nevertheless, effect sizes are generally small to moderate and vary across cognitive domains and intervention protocols, while evidence for transfer to untrained outcomes and everyday functioning remains limited and inconsistent [4, 7].These limitations highlight the need for strategies capable of amplifying training-induced plasticity, promoting transfer beyond the practiced tasks, and potentially achieving clinically relevant effects with a shorter training period.

Non-invasive transcranial electrical stimulation has been proposed as a potential adjunct to DCT [8, 9]. Transcranial alternating current stimulation (tACS) applies sinusoidal electrical currents that can interact with endogenous neural oscillations and modulate task-relevant network dynamics with initial findings indicating significant improvements in auditory verbal learning and delayed recall scores compared to sham stimulation [10, 11]. This approach may be particularly suitable for concurrent administration with DCT because stimulation is delivered while the targeted neural systems are actively engaged, potentially facilitating learning-related plasticity. Moreover, tACS offers the possibility of modulating theta-band oscillations (~ 4–7 Hz), a frequency range closely related to memory processes, cognitive control, and episodic-memory encoding and retrieval [12, 13], aligning external stimulation with the brain’s intrinsic rhythms to enhance cognitive processing. Recent sham-controlled evidence in MCI also suggests potential benefits of multi-site theta-tACS combined with working memory training, with reported improvements in recognition memory and verbal fluency [14].

One of the primary regions targeted in neuromodulation studies is the dorsolateral prefrontal cortex (DLPFC), a central hub for executive control and higher-order cognitive processing [15]. In addition to its well-established role in working memory, the DLPFC contributes critically to the preparation and inhibition of saccadic eye movements, making antisaccade performance a sensitive functional marker of prefrontal engagement and inhibitory control [16, 17]. Because both executive dysfunction and memory impairment are prominent features of MCI, modulation of DLPFC activity may influence not only inhibitory control but also broader cognitive domains relevant to daily functioning.

Computational modeling studies indicate that age-related atrophy and individual differences in head anatomy can influence the strength and distribution of cortical electric fields, providing a rationale for investigating higher stimulation intensities in older adults [18, 19]. However, their tolerability during concurrent cognitive training requires evaluation. Recent clinical guidance describes a generally favorable tolerability profile for tACS while emphasizing continuous monitoring of participant responses and careful inspection of the electrode–skin interface [20]. We therefore selected 3.2 mA peak-to-peak within the low-intensity tES framework described in existing safety guidance [21].

The present pilot study examined whether combining tACS with Digital Cognitive Training (DCT) is feasible and well tolerated in older adults with MCI. The primary outcomes were adherence, acceptability, and adverse effects. Because stimulation targeted the dorsolateral prefrontal cortex (DLPFC), a region critically involved in inhibitory control, we included antisaccade performance as an exploratory functional measure of prefrontal engagement. We further examined whether active stimulation could facilitate transfer effects to broader cognitive domains commonly impaired in MCI, including verbal memory, visual memory, and visuoconstructive abilities. Together, these pilot data are intended to inform a subsequent randomized trial and assess the potential of this low-cost, scalable intervention for cognitive rehabilitation in aging.

## Materials & methods

### Participants

A total of 21 older adults were initially recruited for this pilot study, comprising an active intervention cohort (*n* = 11) and a control cohort (*n* = 10). To enable a comparative exploratory analysis, the control group consisted of the first 10 participants randomized to the Sham tACS arm of our ongoing randomized controlled trial (ClinicalTrials.gov: NCT06733714). Recruitment procedures, the multidisciplinary evaluating team, and all inclusion and exclusion criteria were identical for both groups.

Eligibility criteria for participation included: (a) individuals aged 60 years or older; (b) a clinical diagnosis of MCI according to DSM-5-TR criteria; (c) normal or corrected vision and/or hearing; (d) willingness to participate in the research and agreement to the informed consent form; and (e) Portuguese as the primary language. Exclusion criteria included: (a) untreated neurological or psychiatric disorders; (b) IQ < 80; (c) presence of implanted metallic devices; (d) history of unexplained syncope or seizures; or (e) any contraindications to transcranial electrical stimulation.

Participants were recruited at the Memory Clinic in the Institute of Psychiatry at the *Universidade Federal do Rio de Janeiro* and received multidisciplinary assessments from September 2023 to June 2024. Evaluations included Montreal Cognitive Assessment (MoCA) [22], Wechsler Abbreviated Scale of Intelligence (WASI) [23], Geriatric Depression Scale (GDS) [24], Geriatric Anxiety Inventory (GAI) [25], Basic and Instrumental Activities of Daily Living scales (Katz and Lawton) [26, 27] and medical appointments. The MCI diagnosis was made under the DSM-5-TR criteria, following discussions between a qualified researcher/clinician and by a board-certified psychiatrist (R.P.).

Written informed consent in accordance with the Declaration of Helsinki [28] was provided, and the study protocol was approved by the Ethics Committee of Psychiatry Institute of UFRJ (CAAE 38114820.3.0000.5263).

### Study design

This was a pilot study with an exploratory sham comparator, designed primarily to assess the feasibility and tolerability of combining tACS with Digital Cognitive Training in older adults with MCI, while also exploring preliminary between-group differences. The assessment of participants occurred twice: one week prior to the commencement of the intervention protocol and immediately after its conclusion. The protocol consisted of daily, 30-minute sessions delivered over five consecutive days.

At the end of the intervention, participants in the sham comparator cohort, drawn from the ongoing RCT, were asked whether they believed they had received real or sham stimulation and rated their confidence in this judgment from 0 to 10. This questionnaire was not administered to the open-label active tACS cohort. For the present analysis, the sham participants’ allocations were accessed only after they had completed all study procedures, by a researcher who did not administer tests or outcome assessments.

This pilot study was conducted in preparation for the clinical trial, which is registered on ClinicalTrials.gov under the identifier NCT06733714.

### Intervention

#### Transcranial alternating current stimulation (tACS)

Neuromodulation was administered using a Microestim Foco Research by NKL (NKL, Brasil, ANVISA registration number: 80191680008). Electrode placement followed the 10–20 EEG system, with one electrode positioned over the left dorsolateral prefrontal cortex in the F3 region and the other over the right prefrontal region (FP2). To enhance safety and comfort, impedance was maintained below 5 kΩ, and stimulation was delivered via 5 × 5 cm saline‑soaked sponges.

For the active condition, stimulation parameters were set at a current intensity of 3.2 mA (peak-to-peak) and a frequency of 6 Hz, targeting the left DLPFC. This intensity was selected to increase the likelihood of inducing physiologically relevant cortical electric fields in older adults, in whom age-related anatomical changes, such as cortical atrophy and increased cerebrospinal fluid volume, may attenuate the intracranial field generated by a given scalp current.

Although the amplitude utilized is slightly above the commonly used range (1–2 mA), it remains within the low‑intensity (< 4 mA) described in current safety guidelines for transcranial electrical stimulation. Thus, 3.2 mA was chosen as a mechanistically motivated compromise between field-strength optimization and tolerability/safety. The active stimulation was delivered over a 30-minute session, with gradual 2-minute ramp-in and ramp-out periods to improve comfort and minimize abrupt sensory changes.

For the Sham (control) condition, the device was programmed to mimic the initial sensations of active stimulation. The stimulation involved an initial 2-minute ramp-up to the target intensity followed by an immediate ramp-down, mimicking the initial tactile sensations (e.g., tingling or itching) associated with the active protocol. Following this brief period, the device provided no active current (0 mA) for the remainder of the 30-minute session. In both conditions, participants were continuously monitored for adverse events.

#### Digital cognitive training (DCT)

Concurrently with tACS, participants from both groups engaged in a DCT program designed to stimulate memory, attention and visual processing speed. The training was delivered via BrainHQ platform (from the Posit Science Inc), featuring two tasks, 15 min each.

The first exercise, “Eyes on Details”, aims to improve rapid saccadic eye movements and the ability to perceive subtle visual details. Participants were briefly presented with a series of 3 to 5 images, displayed one at a time in different positions on the screen. Among these images, some are identical while others are merely similar. The task requires participants to identify the locations where the identical images appeared. As they progress, the images are presented more rapidly, increasing the demand on visual processing speed and attentional focus.

The second, “Juggle Factor”, challenges working memory and executive functions by requiring simultaneous information retention and processing. Participants observe a sequence of numbers moving along circular paths on the screen. Their task is to reconstruct the sequence in both the correct numerical order and spatial positions. As participants demonstrate improved performance, the sequences lengthen, the trajectories become more complex, and the speed of movement increases. This exercise enhances the ability to make quick decisions and solve problems in complex, dynamic environments by taxing cognitive flexibility and processing efficiency.

Progress in the training exercises were adjusted to each participant’s performance i.e. task difficulty increased following improvement in participants performance in each exercise, employing an algorithm to maintain individual success rates at approximately 85%. Engaging visual and auditory enhancements, along with the accumulation of stars, incentivized correct performance.

The platform used for DCT provides detailed performance data, including the number of levels completed, the initial and final percentiles relative to the general population, and percentiles adjusted for the participant’s age group. These metrics allow for a comprehensive evaluation of individual progress and the effectiveness of the training over time.

### Procedures

Participants were first evaluated at the laboratory and, in the subsequent week, they started the protocol, receiving tACS plus Digital Cognitive Training (tACS + DCT) for five consecutive days. Each daily session included electrode placement for stimulation through a pair of saline-soaked (0.9% NaCl) surface sponge electrodes (5 × 5 cm) and tACS application for 30 min (with 2 min ramp-up and ramp-down) while performing Digital Cognitive Training in a Tablet. Post-Intervention evaluation was conducted in the following week after completion of the combined tACS + DCT or Sham + DCT sessions.

### Outcome measures

#### Primary outcomes

After each session, participants completed a self-reported questionnaire rating the potential side effects [29] on a numeric scale from 0 to 10 to assess tolerability. The side effects evaluated were: headache, neck pain, scalp pain, tingling sensations, itching, burning sensations, feeling of alertness, sleepiness, difficulty concentrating, mood fluctuation and phosphenes.

In case of any adverse event or intolerable side effect, the session would be interrupted and a score of 10 would be assigned to the scale. We considered 0 meaning no side effects felt and from 1 to 9 tolerable, dividing between mild (1 to 5) and moderate (6 to 9).

Feasibility and tolerability criteria followed guidelines for pilot trials [30]. Success was defined as adherence ≥ 80% of planned sessions, attrition ≤ 20%, data completeness ≥ 90% of planned assessments and tolerability defined as ≥ 80% of sessions with only mild or moderate side effects and no serious adverse events.

#### Secondary outcomes

We also aimed to analyze the preliminary effects of tACS + DCT on oculomotor behavior and cognition.

Oculomotor Measures:

- Experimental apparatus

The experiments were created and presented using the Experiment Builder Software (SR Research Ltd.). Stimuli were presented on a 24-inch LCD monitor (Philips 144 Hz refresh rate and 1920 × 1080 pixels resolution). A video-based eye tracker (Eyelink1000 plus, SR Research Ltd., Ontario, Canada) with a sampling rate of 1000 Hz was used for monocular recording of the right eye and the participant’s head was stabilized by a chin and forehead rest that enabled the viewing distance between the eyes and monitor to be fixed at 57 cm.

- Antisaccade task

Oculomotor assessment was included as an exploratory behavioral probe of inhibitory control, a cognitive process strongly dependent on prefrontal circuitry. We therefore focused on antisaccade performance, because this task requires participants to suppress a reflexive eye movement toward a peripheral stimulus and instead generate a voluntary saccade in the opposite direction.

Each trial started with the presentation of a central fixation point on a black background (diameter: 0.5°; luminance: 42 cd/m²; background luminance: 0.1 cd/m²). Participants were instructed to maintain fixation and, after the appearance of a peripheral target, to look as quickly and accurately as possible in the opposite direction. After a central fixation period of 1,000 ms, the fixation point disappeared for 200 ms, followed by the appearance of a white peripheral dot (diameter: 0.5°; luminance: 42 cd/m²) for 1,000 ms, presented either to the left or to the right of fixation at an eccentricity of 10° along the horizontal axis (see Supplementary Fig. 1). Each trial included a 1,000 ms inter-trial interval.

The antisaccade task comprised 60 trials, with left- and right-sided target locations presented in randomized order. The main antisaccade outcomes were reaction time, number of omissions, and total errors. Reaction time was used as an index of response speed, whereas omissions and total errors were interpreted as indicators of oculomotor inhibitory-control performance.

#### Neuropsychological measures

Global cognitive function was assessed using the Montreal Cognitive Assessment (MoCA) (score range: 0–30), an instrument that evaluates attention, executive functions, memory, language, visuospatial abilities, and temporal and spatial orientation [22]. Intellectual quotient (IQ) was measured with the Wechsler Abbreviated Scale of Intelligence (WASI), which comprises four subtests [23].

To assess verbal learning and memory, we employed the Rey Auditory Verbal Learning Test (RAVLT) [31], a widely used measure of episodic verbal memory that evaluates the processes of encoding, consolidation, storage, and retrieval of verbally learned material, is a widely used measure of episodic verbal memory that assesses the encoding, consolidation, storage, and retrieval of verbally learned material [32]. Visuoconstructive abilities and visual memory were evaluated using the Rey–Osterrieth Complex Figure Test (ROCF) [33] which also provides indicators of executive functions related to planning, perceptual organization, and visual processing strategies. Additional information on each instrument is provided in the supplementary material.

### Statistical analysis

Statistical computations were performed using R software (R Foundation for Statistical Computing, Vienna, Austria) alongside GraphPad Prism version 8.0.2 (GraphPad Software, San Diego, CA, USA). Initially, the normality of the data distributions was assessed using the Shapiro-Wilk test.

To ensure comparability between the active tACS and Sham groups prior to the intervention, baseline demographic and clinical characteristics were compared. Independent two-tailed t-tests were used for normally distributed continuous variables (e.g., age, years of education). For ordinal data or variables that violated normality assumptions, including clinical and functional scales (e.g., MoCA, GDS, GAI, Katz, Lawton), the non-parametric Mann-Whitney U test was applied.

To explore the preliminary efficacy of the intervention on cognitive and oculomotor performance, we conducted a 2 × 2 Mixed Analysis of Variance (ANOVA). In this model, *Time* (Pre-intervention vs. Post-intervention) was entered as the within-subjects repeated measure factor, and *Group* (Active tACS vs. Sham) was entered as the between-subjects factor. The primary metric of interest for differential efficacy was the *Time × Group* interaction, which evaluates whether the trajectory of cognitive or oculomotor changes differed significantly between the two groups.

Bonferroni correction was applied to the two within-group pre–post comparisons for each outcome. No adjustment was applied across the secondary outcome battery, and ANOVA p-values are reported as unadjusted exploratory results.

Furthermore, to explore whether the subjective experience of tACS side effects was associated with engagement in the Digital Cognitive Training (DCT), a Spearman rank-order correlation analysis was conducted. The number of DCT levels completed served as the outcome variable, and the mean self-reported side-effect score per session served as the predictor. Considering the slightly higher current intensity used in this pilot (3.2 mA), this analysis was performed at the session level to assess whether a greater side-effect burden impaired DCT performance.

Blinding was assessed within the sham comparator cohort using Bang’s Blinding Index, with a 95% confidence interval obtained by transforming the exact binomial confidence interval for the proportion of correct guesses. Confidence ratings were summarized descriptively. To explore sensory symptoms in relation to allocation guesses, participant-level mean phosphene and tingling intensity scores were calculated across available sessions, without imputing missing observations, and summarized by guessed allocation.

## Results

### Sample characterization

A total of 21 older adults with a clinical diagnosis of MCI were initially enrolled in the study. One participant from the active intervention cohort was excluded due to an IQ score below 80, resulting in a final sample of 20 participants who successfully completed the protocol (10 assigned to the active tACS group and 10 to the Sham group).

The cohorts were predominantly female (80% in both arms). As detailed in Table 1, the active tACS and Sham groups were well-matched at baseline. There were no statistically significant differences between the groups regarding demographic characteristics such as age (*p* = 0.34) and years of education (*p* = 0.65). Furthermore, clinical and functional profiles were highly comparable, with no significant differences in global cognition (MoCA, *p* = 0.44), depressive symptoms (GDS, *p* = 0.51), basic activities of daily living (Katz, *p* = 0.30), or instrumental activities of daily living (Lawton, *p* = 0.36). A marginal trend toward a difference was observed only in baseline anxiety symptoms (GAI, *p* = 0.059).

**Table 1:** Demographic and clinical information

Subjects characteristics	tACS (*n* = 10)	Sham (*n* = 10)	*p*
Age (years)	71.00 (7.40)	68.10(7.68)	0.34
Education (years)	14.10 (3.38)	13.40(3.47)	0.65
MoCA	22.60 (3.40)	23.10(4.43)	0.44
Sex M: F	2:8	2:8	-
GDS	4.30 (2.57)	3.70 (2.54)	0.51
GAI	3.50 (3.40)	5.60 (3.47)	0.06
Katz	5.90 (0.31)	5.70 (0.48)	0.30
Lawton	26.30 (1.05)	26.80 (0.42)	0.36

Mean(SD), MoCA: Montreal cognitive assessment (0–30 points), IQ: intellectual quotient (80–160 points), M: Male, F: Female. GDS: Geriatric Depression Scale (0–15 points), GAI: Geriatric Anxiety Inventory (0–20 points), Katz: Basic Activities of Daily Living scale (0–6 points), Lawton: Instrumental Activities of Daily Living scale (9–27 points)

### Feasibility and tolerability

Participants in the active tACS + DCT group showed excellent adherence to the protocol, with no dropouts or missed sessions, despite receiving no financial stimulus for participation. All 10 participants completed the five-session intervention, yielding 50 active tACS + DCT sessions for analyses. This provided a robust basis for characterizing adherence and self-reported side effects in the active stimulation condition. Descriptive feasibility data from the sham comparator group are presented to contextualize these findings.

All feasibility and tolerability criteria were met or exceeded: adherence was 100% (surpassing the ≥ 80% threshold) and attrition 0% (below the ≤ 20% threshold), and data completeness 100% of planned assessments. Across the 50 sessions, 90% met the tolerability criterion of mild or moderate sensations with no serious adverse events.

In the active tACS group, 67% of sessions were associated with mild side effects and 23% with moderate side effects. No severe side effects or adverse events were observed, and all sessions were completed without interruption. The most frequently reported symptoms during active stimulation were tingling under the electrode (34% of sessions; mean intensity 1.4/10) and phosphenes (22%; mean intensity 5.0/10), whereas the remaining symptoms were less frequent and generally reported at low average intensity.

Self-reported symptoms were also common in the sham group. The most frequently reported symptoms in this condition were phosphenes (50%; mean intensity 5.04/10), tingling under the electrode (45%; 3.81/10), difficulty concentrating (39%; 3.31/10), feeling of alertness (39%; 5.15/10), and headache (31%; 4.26/10). Overall, this descriptive pattern indicates that subjective sensations were not restricted to active stimulation and were also frequently reported during sham sessions (Table 2).

**Table 2:** tACS Side effects

Self-reported symptoms	tACS		Sham	
	Frequency of occurrence (%)	Average intensity (0–10)	Frequency of occurrence (%)	Average intensity (0–10)
Tingling under the electrode	34%	1.4	45%	3.81
Phosphenes	22%	5.0	50%	5.04
Difficulty concentrating	18%	2.1	39%	3.31
Headache	16%	2.5	31%	4.26
Feeling of alertness	14%	1.0	39%	5.15
Itching	12%	1.8	14%	4.42
Burning under the electrode	8%	4.0	8%	5.25
Neck pain	8%	2.2	16%	4.37
Drowsiness	8%	2.0	22%	2.54
Mood fluctuation	4%	0.5	6%	6.3
Pain under the electrode	2%	4.0	10%	4.4

Self-reported side effects felt during the tACS sessions; Frequency (%) and average intensity (0–10); transcranial alternating current stimulation (tACS)

Among the ten sham participants, eight believed they had received active stimulation, with a mean confidence rating of 8.25 (SD 2.12), whereas two correctly identified sham stimulation, with a mean confidence rating of 5.50 (SD 6.36). Bang’s Blinding Index was − 0.60 (exact 95% CI − 0.95 to 0.11), indicating predominantly opposite guesses. Participant-level mean phosphene and tingling intensity scores were descriptively higher among those who guessed active stimulation than among those who guessed sham, with mean differences of 1.50 and 2.32 points, respectively, on the 0–10 scale. With only two participants identifying sham, these comparisons provide limited information about the relationship between sensory symptoms and allocation guesses.

Participants in the active tACS group completed 45.8 ± 6.5 levels in the Digital Cognitive Training whereas participants in the Sham comparator group completed 48.7 ± 8.6 levels. To examine whether side effects during active tACS were associated with Digital Cognitive Training performance, we computed a Spearman rank-order correlation between the mean side-effect score for each session and the number of DCT levels completed in each session in the active tACS group (number of sessions = 50). The correlation was small and non-significant (rₛ = 0.18, 95% CI [-0.12, 0.44], *p* = 0.22), providing no evidence that higher side-effect ratings were linked to reduced training performance.

Given that most reported side effects were mild to moderate, these findings support the tolerability of the combined tACS + DCT protocol and suggest that the slightly higher current intensity used in (3.2 mA) did not measurably impair participants’ engagement in Digital Cognitive Training.

### Exploratory analysis of oculomotor inhibitory control

In an exploratory analysis, we evaluated inhibitory control using the antisaccade task, which requires suppressing a reflexive glance toward a peripheral stimulus and looking in the opposite direction instead. This function depends strongly on the dorsolateral prefrontal cortex (DLPFC), the stimulation target (Table 3).

**Table 3:** Exploratory inhibitory control assessment via antisaccade outcomes before and after five sessions of active tACS + DCT and sham + DCT in older adults with MCI

	tACS pre	tACS post	Sham pre	Sham post	tACS post–pre *p*	Sham post–pre *p*	Time effect F, *p*	Group x Time interaction F, *p*
Total errors	15.11 (3.59)	10.22 (3.73)	15.29 (4.03)	16.80 (3.80)	0.04	0.93	1.57, 0.23	5.98, 0.03
Reaction time	93.02 (19.77)	74.78 (16.16)	112.40 (38.72)	88.42 (21.71)	0.21	0.15	**7.74**,** 0.01**	0.14, 0.71

Values are presented as mean (SD). Repeated-measures ANOVA was performed with group (active tACS + DCT vs. Sham + DCT) as the between-subject factor and time (pre vs. post) as the within-subject factor. Post hoc within-group Post–Pre comparisons were performed using paired t-tests, with Bonferroni correction applied within each outcome to account for the two group-specific comparisons. Lower values indicate better performance for total errors and lower reaction time indicates faster responses. Bold indicates *p* < 0.05

**Fig. 1:**
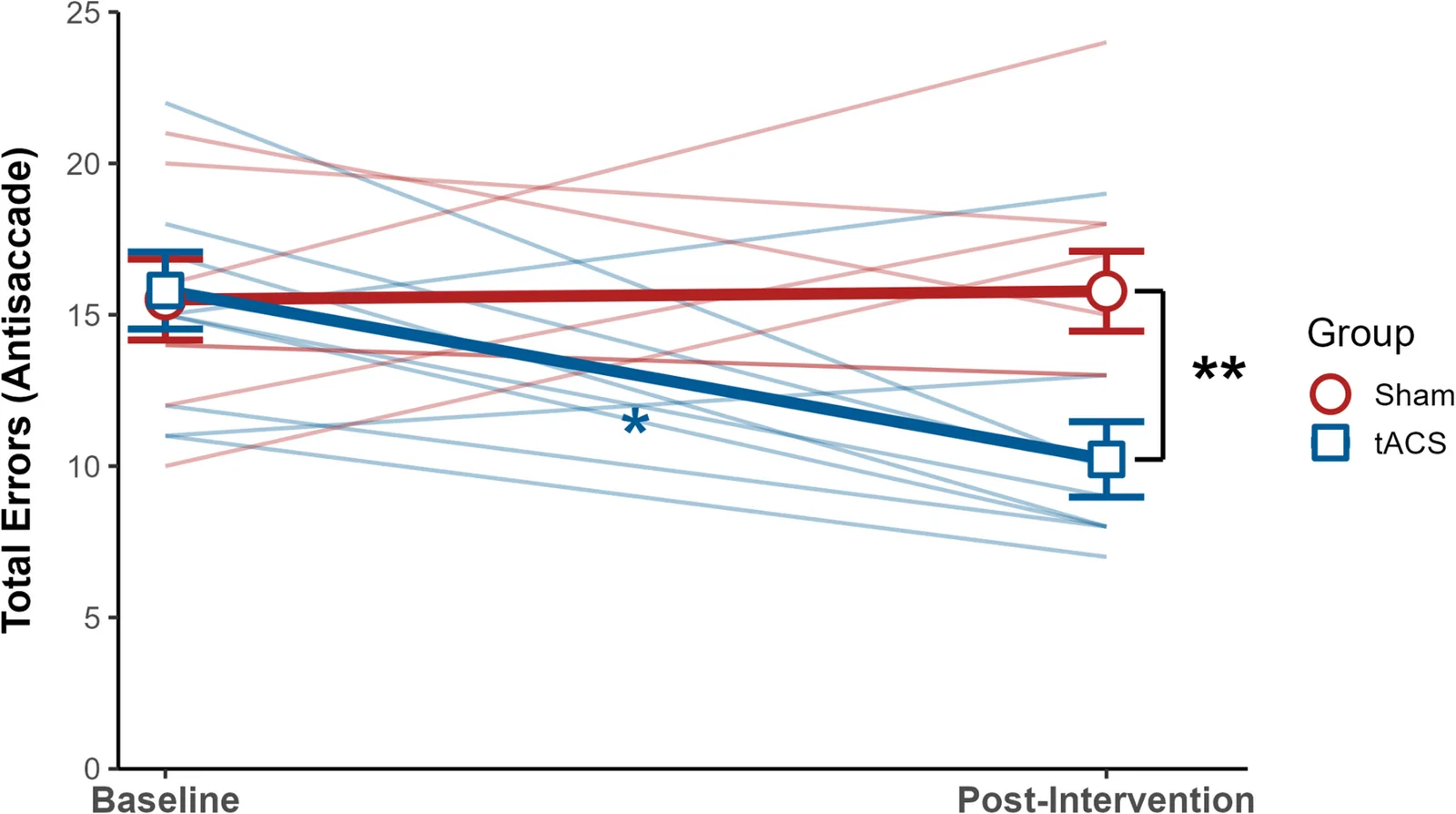
Exploratory effects of active tACS + DCT versus sham + DCT on ANTISACCADE task total errors in older adults with MCI. Exploratory effects of active tACS + DCT versus sham + DCT on antisaccade task total errors in older adults with mild cognitive impairment (MCI). Values are shown as mean ± standard error (SE) at baseline (pre) and post-intervention. Repeated-measures ANOVA revealed a significant group × time interaction for antisaccade total errors (F = 5.98, *p* = 0.03), indicating a greater reduction in total errors in the active tACS + DCT group than in the sham + DCT group. Post hoc Bonferroni-corrected comparisons showed that antisaccade total errors decreased significantly from pre- to post-intervention in the active group (*p* = 0.04), but not in the sham group (*p* = 0.93). The groups did not differ at baseline (*p* = 0.92), but differed significantly at post-intervention (*p* = 0.008), with fewer antisaccade errors in the active stimulation group. Lower values indicate better inhibitory control. *p < 0.05 for within-group change in the active condition; **p < 0.01 for between-group difference at post-intervention

Participants in the active tACS + DCT condition showed a significant reduction in antisaccade errors (from approximately 15 to 10 errors; *p* = 0.04), whereas the sham + DCT group showed no significant change (*p* = 0.93) (Fig. 1). Although both groups performed comparably at baseline, a clear between-group divergence emerged following the intervention, favouring the active stimulation condition. This differential change was corroborated by a significant group × time interaction (Table 3; Fig. 1).

Antisaccade reaction time, which mainly reflects processing speed and response preparation rather than inhibitory control itself, became faster over time across participants, with no significant pre–post change within either group (tACS: *p* = 0.21; sham: *p* = 0.15) and no difference between groups. This suggests a general practice or training-related effect that was not specific to active stimulation (Table 3).

Overall, these exploratory findings provide preliminary evidence that active tACS combined with DCT may improve oculomotor inhibitory control, particularly by reducing antisaccade total errors. This effect was specific to the active stimulation group and emerged despite comparable baseline performance, although it should be interpreted cautiously given the pilot design and small sample size.

### Cognitive performance

An important goal of cognitive interventions in MCI is not only to improve performance on trained tasks but also to promote transfer effects to broader cognitive domains affected by the disorder. Therefore, we explored whether combining tACS with DCT produced greater gains than sham + DCT in neuropsychological measures of verbal memory, visual memory, and visuoconstructive abilities, domains frequently impaired in MCI (Table 4).

**Table 4:** Exploratory cognitive outcomes before and after five sessions of active tACS + DCT or sham + DCT in older adults with MCI

Cognitive domain	tACS pre	tACS post	Sham pre	Sham post	tACS post–pre *p*	Sham post–pre *p*	Time effect F, *p*	Group x Time interaction F, *p*
Visuoconstructive abilities	0.36 (0.54)	0.80 (0.50)	0.63 (0.75)	0.63 (0.97)	0.05	1.00	3.94, 0.06	4.24, 0.05
Verbal Memory	-0.54 (1.41)	-0.01 (1.19)	-0.22 (1.09)	0.70 (2.07)	0.06	0.27	**7.22**,** 0.01**	0.52, 0.47
Visual memory	0.19 (1.40)	0.72 (1.44)	0.75 (1.31)	1.35 (1.59)	0.09	**0.03**	**13.90 < 0.01**	0.04, 0.83

Values are presented as mean (SD). Analyses were conducted using complete cases for each outcome. Repeated-measures ANOVA was performed with group (active tACS + DCT vs. sham + DCT) as the between-subject factor and time (pre vs. post) as the within-subject factor. The Time effect and Group × Time interaction columns report F statistics and unadjusted ANOVA p-values. Within-group Post–Pre comparisons were performed using paired t-tests, with Bonferroni correction applied within each outcome to account for the two group-specific comparisons. Bold indicates *p* < 0.05. RAVLT: Rey Auditory Verbal Learning Test; ROCF: Rey–Osterrieth Complex Figure Test

Visuoconstructive abilities (ROCF copy) showed a trend-level group × time interaction (Table 4), suggesting greater improvement in the active tACS + DCT group compared with the sham + DCT group. Consistent with this finding, post-hoc analyses showed a trend-level pre–post improvement in the active tACS + DCT group (*p* = 0.05), whereas no change was observed in the sham + DCT group (*p* = 1.00). A trend-level main effect of time was also detected (Table 4).

Verbal memory (RAVLT total learning score (A1–A5)) showed a significant main effect of time (Table 4), indicating overall improvement across participants. Although the group × time interaction was not significant, the active tACS + DCT group demonstrated a trend toward improvement (*p* = 0.06), whereas the sham + DCT group did not (*p* = 0.27).

Visual memory (ROCF recall) also improved over time, with no evidence of a stimulation-specific effect, as the group × time interaction was not significant (Table 4). Bonferroni-corrected comparisons showed a significant pre–post improvement in the sham group (*p* = 0.03), but not in the active group (*p* = 0.09).

Overall, these exploratory analyses provide preliminary evidence that active tACS may enhance transfer effects to selected cognitive functions. Although robust stimulation-specific effects were not observed across all neuropsychological measures, the pattern of results suggests potential cognitive benefits that warrant investigation in adequately powered studies.

## Discussion

This pilot study evaluated the feasibility and tolerability of combining tACS with Digital Cognitive Training in older adults with MCI and explored cognitive and oculomotor outcomes using a sham comparator. All participants in both groups completed the five-session protocol, with no missed sessions or dropouts, despite the absence of financial compensation. This high adherence supports the feasibility of delivering the combined intervention in this population.

Regarding tolerability, self-reported symptoms during active stimulation were predominantly mild to moderate (Table 2), and no severe side effects or adverse events (i.e., any unintended and harmful medical occurrence) were reported during the study. The profile observed in the active tACS group—including tingling under the electrode, transient phosphenes, occasional headache, difficulty concentrating, and itching—is consistent with established evidence that tACS-related adverse effects are typically mild and short-lived, including in older adults [34].Reports of phosphenes during frontal or occipital tACS are well documented and are generally interpreted as transient neurosensory phenomena rather than indicators of serious toxicity [35].

Notably, self-reported symptoms were also common in the sham group, and some were descriptively more frequent or intense than in the active condition. This pattern suggests that subjective sensations were not restricted to active stimulation and may partly reflect non-specific factors, such as expectancy, procedural context, or affective responses to repeated sessions. A tendency toward greater anxiety-related or attentional complaints in the sham group may also have contributed to symptom reporting, although this interpretation remains speculative. In addition, because the sham participants were drawn from the blinded arm of the ongoing RCT whereas the active participants came from an open-label pilot cohort, differences in blinding and study context may have influenced how symptoms were perceived and reported. These descriptive between-group differences should therefore be interpreted cautiously and should not be taken as evidence that sham stimulation was less tolerable than active tACS.

Our findings align with previous research, which indicates that tACS exhibits a favorable tolerability profile [29] and that Digital Cognitive Training can achieve high adherence rates in older adults [36]. Moreover, framing feasibility and tolerability as primary outcomes aligns with pilot-trial guidance that emphasizes pre-specified “progression criteria” (e.g., adherence/retention, data completeness, and adverse-event thresholds) to judge readiness for a definitive trial [37]. Our exploratory Spearman rank-order correlation between session-level side-effect burden and the number of Digital Cognitive Training levels completed adds a novel contribution by suggesting that subjective sensations during active tACS were not associated with poorer engagement in the training task. These findings support the feasibility of integrating tACS and DCT within the same session, although they should not be interpreted as evidence of synergistic efficacy.

Exploratory neuropsychological outcomes showed time-related changes in selected domains, but did not provide robust evidence of superiority of active stimulation over sham. Verbal memory and visual memory improved over time, whereas group × time interactions were not significant, suggesting that practice or retest effects may have contributed to these changes. In contrast, visuoconstructive abilities showed a trend-level group × time interaction, with a trend-level pre–post improvement in the active tACS + DCT group and no corresponding change in the sham + DCT group. These findings should therefore be interpreted as preliminary signals rather than evidence of efficacy, although they are compatible with literature showing that theta-tuned or cross-frequency tACS can modulate memory-relevant dynamics in aging, though effects are heterogeneous across protocols, outcomes, and populations [38]. A novel aspect of this study was the inclusion of oculomotor behavior analysis, which served as an indicator of DLPFC functionality and an indirect measure of cognitive processes. This interpretation is consistent with evidence implicating the dorsolateral prefrontal cortex in antisaccade control and executive inhibition [16].

The oculomotor analysis was framed around inhibitory control, using antisaccade performance as an exploratory behavioral probe of prefrontal function. This rationale is particularly relevant because the antisaccade task requires suppression of a reflexive eye movement toward a peripheral stimulus and generation of a voluntary saccade in the opposite direction. Within this framework, the clearest signal was observed for antisaccade total errors, which showed a significant group × time interaction, driven by a reduction in errors in the active tACS + DCT group but not in the sham + DCT group. Reaction time improved over time across groups, suggesting a non-specific time or practice effect, whereas omissions showed only a trend-level group × time interaction. Taken together, these findings suggest a possible differential effect of active stimulation on oculomotor inhibitory control, a function consistently linked to prefrontal circuitry [17]. Although exploratory and based on a small sample, the reduction in antisaccade errors relative to sham may support the functional relevance of targeting the left DLPFC for goal-directed behavior and executive control.

The current literature on tACS for cognitive enhancement exhibits significant heterogeneity in terms of stimulation parameters, including frequency, current intensity, and electrode positioning. While some studies have used gamma or alpha frequencies [39], our choice of 6 Hz theta frequency aligns with evidence supporting its role in memory encoding and retrieval [40]. Within this heterogeneous landscape, modest amplitude increases in older adults are mechanistically justified by dose–field dependencies and age-related anatomy (e.g., increased CSF thickness, cortical atrophy) that attenuate intracranial electric fields for a given scalp current; entrainment at the neuronal level has been observed at field strengths on the order of tenths of V/m in primate and human work [19, 41]. The targeted stimulation of the DLPFC, a region implicated in working memory [15] and saccadic eye movements [42], further emphasizes the importance of precise protocol design to monitor its neurophysiological effects.

Despite being a pilot study, several limitations must be acknowledged. First, the small sample size and the exploratory nature of the between-group comparison limit the generalizability of our findings. Although the secondary outcome battery was limited and selected on theoretical grounds, no multiplicity adjustment was applied across outcomes, increasing the risk of false-positive findings. These results should therefore be regarded as hypothesis-generating rather than confirmatory evidence of efficacy. Second, the active and sham cohorts were not recruited and randomized within a unified parallel-arm design. The active cohort was open-label, whereas the sham comparator comprised participants from the ongoing blinded RCT. Despite shared eligibility criteria and intervention procedures, differences in participant selection, treatment expectations, and study context may have influenced the observed outcomes. The between-cohort comparisons should therefore be considered strictly preliminary and cannot establish a causal effect of active stimulation. Blinding assessment was available only for the sham cohort, further limiting evaluation of these potential influences. The small sham sample and the predominance of active-stimulation guesses also limited assessment of whether sensory symptoms influenced perceived allocation. Third, cognitive outcomes were reassessed over a short interval (within approximately two weeks) and without alternate test forms, so learning or practice effects represent an important potential confound, particularly for visuoconstructional measures such as the Rey–Osterrieth Complex Figure. Alternate versions were not used because, to our knowledge, they have not been validated for the Brazilian older adult population, and non-validated forms could compromise the comparability of scores between time points. Fourth, the short duration of the intervention (five consecutive days) may not capture the full potential of a longer-term protocol. Another potential limitation is that participants had a relatively high level of education, which may constrain the generalizability of the findings to the broader population. Lastly, the reliance on self-reported measures of side effects may introduce bias, as participants could have under- or over-reported their experiences.

Besides its limitations, this pilot integrates mechanism-guided theta-tACS, an age-aware dosing rationale, and Digital Cognitive Training with oculomotor behavior biomarkers—providing a feasible, tolerable, low-cost avenue for cognitive rehabilitation in MCI. The high adherence rates, robust performance on DCT despite typical tACS sensations, and absence of severe side effects underscore the practicality of our approach, while the integration of both neuropsychological and oculomotor behavior measures provides a comprehensive assessment of intervention effects. Critically, feasibility and tolerability outcomes at a slightly higher intensity supported progression to a definitive trial. Building on these promising preliminary findings, our pilot study lays the foundation for a future double-blind, randomized controlled trial (NCT06733714, ClinicalTrials.gov) to evaluate the efficacy of the combined tACS + DCT intervention as a viable therapeutic option for cognitive enhancement in MCI.

## Conclusion

In summary, this pilot study demonstrated that combining tACS and Digital Cognitive Training in older adults with MCI is both feasible and well-tolerated, even when applying a slightly higher stimulation intensity (3.2 mA) within low-intensity transcranial electrical stimulation bounds. Preliminary cognitive and oculomotor findings, consistent with DLPFC engagement, lead us to speculate that the combined intervention may benefit cognitive performance in individuals with MCI, even though these signals require confirmation in controlled designs. Meeting our pre-specified feasibility and tolerability thresholds supported progression to a definitive trial, and a double-blind randomized controlled study has been initiated to test efficacy on neuropsychological outcomes and oculomotor behavior (ClinicalTrials.gov: NCT06733714).

Future research should build on these results by employing larger sample sizes, double-blind randomized controlled designs, and longer intervention durations. Exploring alternative stimulation frequencies, durations, and electrode placements could further refine the protocol. Additionally, studies assessing the long-term cognitive and neurophysiological impacts of this combined intervention are warranted. Collectively, these insights outline a low-cost, scalable pathway for developing neuromodulation-plus-training strategies to enhance cognitive health in older adults.

## Data Availability

De‑identified individual participant data and outcome measures are available from the corresponding author upon reasonable request for up to five years following publication.
